# Awareness, utility and preferences of campus-based mental health services at tertiary institutions in Harare, Zimbabwe: A cross-sectional study

**DOI:** 10.1371/journal.pgph.0005107

**Published:** 2026-05-06

**Authors:** Omega Mukubvu, Gamuchirai C. Tonono, Ropafadzo L. Mupinga, Chris T. Uriga, Yola A. Machona, Shalom R. Doyce, Edwin Mavindidze, Rufaro H. Mushonga, Beatrice K. Shava, Sidney Muchemwa, Dixon Chibanda, Anotida R. Hove, Jermaine M. Dambi

**Affiliations:** 1 Department of Rehabilitation Sciences, Faculty of Medicine and Health Sciences, University of Zimbabwe, Avondale, Harare, Zimbabwe; 2 Department of Pathology, Kamuzu University of Health Sciences, Blantyre, Malawi; 3 Mental Health Unit, Faculty of Medicine and Health Sciences, University of Zimbabwe, Avondale, Harare, Zimbabwe; University of Cape Town, SOUTH AFRICA

## Abstract

Mental health (MH) disorders are highly prevalent among university students, with multi-level impacts. Although campus-based mental health services (CBMHS) are available, awareness and utilisation rates remain low. This study examined the awareness and utilisation of CBMHS among Zimbabwean tertiary students, including the barriers, facilitators, and preferences that influence their access to services. This descriptive cross-sectional study recruited 1070 students from five tertiary institutions in Harare, Zimbabwe. Participants completed questionnaires evaluating awareness, utilisation, preferences, and barriers to accessing CBMHS. Data were analysed using descriptive statistics and multivariate logistic regression. The mean age of the participants was 21.7 (SD = 2.7) years. 76.5% of students were aware of CBMHS. Awareness of MH services was associated with familial history of MH conditions [AOR = 1.35 (95% CI: 1.05; 1.83), p = .05] and personal experience of a MH condition [AOR = 0.71 (95% CI: 0.52; 0.97), p = .030]. Only 16.5% of students had utilised CBMHS. High utility of CBMHS was associated with drug and substance use [AOR = 2.388 (95% CI: 1.227; 4.644), p = 0.01], availability of a psychologist [AOR = 1.69 (95% CI: 1.10; 2.59), p = .017] and availability of campus-wide MH workshops [AOR = 1.62 (95% CI: 1.00; 2.60), p = .049]. Key barriers to MH service utilisation included lack of resident/institutional MH service providers [AOR = 2.02 (95% CI: 1.16; 3.51), p = .013], past MH experiences [AOR = 1.53 (95% CI: 1.16; 2.02), p = .002], and friend’s history of MH condition [AOR = 1.44 (95% CI: 1.11; 1.86), p = .006]. Students preferred self-help services, individual therapy, and psychologists for MH support. Universities should promote MH awareness through effective awareness campaigns and workshops, provide tailored services that align with the students’ preferences, and ultimately create a supportive environment that fosters students’ overall MH service utilisation and well-being.

## Introduction

Common mental disorders (CMDs), particularly depression and anxiety, are highly prevalent among university students. Approximately 35% of university students experience significant mental health (MH) problems in a lifetime. The beginning of tertiary education signifies a transitional period for young adults, during which they face challenges such as making independent decisions about their personal lives and adapting to increased academic pressures [1]. The transitional period is characterised by an increased risk of CMDs, uncertainties about the future, potentially leading to a cycle of poor MH, and decreased academic performance and social functioning [2–4]. Moreover, in low-resource environments, students who relocate from other places for tertiary education often encounter additional obstacles. These include limited social support and financial hardships, both of which elevate their risk of experiencing poor MH [5]. Therefore, early identification and treatment of CMDs are crucial for students to mitigate the adverse effects [6]. Failure to address CMDs in tertiary students can result in lower academic performance, increased substance use, risky sexual behaviours, and social difficulties. These problems may persist into adulthood, resulting in various psychosocial and economic consequences [7–10]. Consequently, various campus-based mental health (CBMH) interventions, including counselling services, digital interventions, referral pathways for stepped-up care, and health promotional initiatives, have been implemented to improve students’ mental well-being [11,12]. Campus–based mental health services (CBMHS) serve as a crucial entry point for both preventive and curative care. These services (CBMHS) are potentially cost-effective and enhance accessibility to MH services for tertiary students [5,13]. However, several factors, such as the limited availability of specialised professionals, concerns about stigma, and privacy considerations, can affect the uptake and utilisation of CBMHS [14–16]. For instance, a systematic review (N = 44) showed that the utilisation of MH services among universities ranged from 13.7% to 68.7% [14]. However, in this systematic review, only one article was from Sub-Saharan Africa (SSA), with most of the studies (n = 32) originating from the USA [14]. The limited evidence base from the SSA region is concerning, given the historically disproportionate MH treatment gap within the region [5,17].

Generally, the awareness and uptake of MH services in low- and middle-income countries (LMICs) is significantly lower compared to high-income countries (HICs) [5,18,19]. This is often attributed to lower mental health literacy (MHL) thus low knowledge of where to seek help, limited availability of CBMHS, stigma, systemic, structural barriers such as cost, underfunding and competing priorities in LMICs compared to HICs where MHS are more widespread [17,20,21]. Despite the abundance of MHS in HICs this does not equal access; utilisation also remains low due to a lower perceived need and concerns of effectiveness of available interventions [22]. Low awareness and utilisation of the existing CBMHS exacerbate the treatment gap within universities in SSA [5,23]. The key active ingredients for the optimal uptake or utilisation of CBMHS include increased awareness, positive attitudes, and perceptions towards the available MH services. The underutilisation is influenced by factors such as low MHL, stigma, negative attitudes, peer labelling, lack of awareness, gender expectations, academic pressure, cultural beliefs, time constraints, confidentiality concerns, accessibility, low resources, self-reliance, and preference for support from family and friends [5,16,18,24–29]. For example, a Nigerian cross-sectional study (N = 450) reported that 67% of students had never used MH services [30]. The low utilisation was attributed to poor health-seeking behaviours, limited social support, and insufficient information on available MH services [30]. Another cross-sectional study among South African tertiary students (N = 28516) showed that 71.3% reported having an MH problem, yet only half (35.2%) had utilised on-campus MH services [19]. Also, structural factors, including formal CBMHS policy to implementation gaps shape how services are provided and accessed. Evidence from Sub-Saharan Africa indicates that such policy frameworks are often limited, leading to the underutilisation CBMHS [17]. Adolescents and young adults are especially susceptible to CMDs but often hesitate to seek professional help due to various barriers to accessing MH services, such as stigma, negative attitudes towards treatment, and financial barriers [31]. Tertiary students seek MH services when assured of confidentiality, respect, and no judgment [5]. Factors such as positive past experiences, good MHL, encouragement from others, belief in effective treatment and the mode of delivery of MH services also influence engagement with CBMHS [5,13,32,30]. For instance, studies have demonstrated disparities in the preferences for the mode of delivery of MH services. Some students prefer face-to-face over online MH services, while others favour blended, i.e., a combination of both physical and online CBMHS [33,34]. Therefore, it is paramount to understand the MH service preferences of Zimbabwean students to inform the development of targeted MH services or modification of existing services, particularly in the context of the historical prioritisation of sexual and reproductive health (SRH) over MH. Importantly, Zimbabwe has also seen an exponential increase in the burden of CMDs in tertiary students, as in the general population. However, as with other low-resource settings, a substantial treatment gap exists, and there have not been previous attempts to explore the uptake of the available CBMHS in Zimbabwe. Previous studies in Sub-Saharan Africa have largely focused on prevalence and correlates of MH problems rather with a dearth of empirical knowledge on how contextual, attitudinal and structural factors interact to shape engagement with CBMHS. This study, thus, aimed to assess the utilisation and awareness of CBMHS among Zimbabwean tertiary students. We also sought to identify the barriers, facilitators, and preferences of tertiary students regarding the use of CBMHS. It is important to assess the level of awareness and utilisation of CBMHS among Zimbabwean tertiary students, and to identify key barriers, facilitators, and preferences influencing their use. Insights from this study could guide the design of bespoke interventions, including optimising the uptake of existing services to improve the mental well-being of university students from low-resource settings.

## Materials and methods

### Ethics statement

Ethical approval was obtained from the Joint Parirenyatwa & the University of Zimbabwe, Faculty of Medicine and Health Sciences Research and Ethics Committee (JREC) [JREC: 25/2025]. Institutional approval was granted from the five tertiary institutions where the study was conducted. The study adhered to the principles outlined in the Declaration of Helsinki. For instance, written informed consent was obtained from each participant, and they were treated as autonomous agents, as they could withdraw from the study at any time without consequence.

### Study design and setting

This cross-sectional study was conducted from February 26, 2025, to April 28, 2025. Participants were recruited from five tertiary institutions, i.e., the University of Zimbabwe, the Women’s University in Africa, the Harare Institute of Technology, Harare Polytechnic, and the Catholic University of Zimbabwe. The study sites are all located in Harare. Harare was conveniently selected because it is the metropolitan capital of Zimbabwe with a concentration of established tertiary institutions. Harare attracts students from all provinces and produces the most diverse student body in the country. The five institutions included in this study have a combined enrolment of 34,557 students, approximately 30% of all tertiary students in Zimbabwe. Additionally, the universities were selected based on accessibility and geographic proximity to the research team, given that the study was conducted as an unfunded undergraduate research project. Across the institutions, different CBMHS are offered, such as campus-wide MH awareness campaigns, psychotherapies, peer-led interventions, and referrals for stepped-up care.

### Participants

University students were recruited through a multistage sampling process. The first stage involved stratified proportionate sampling, where a proportional sample was selected from each of the five institutions. The stratum size was proportional to the university’s total intake/size relative to the study population therefore researchers determined the required sample size from each institution according to the enrolment. This was done to ensure the sample is a representative of the overall population. Once stratified, university students were conveniently recruited into the study according to students available on the day of data collection. Students were approached during lunchtime in easily accessible spaces, including cafeterias, sports fields, and open seating areas. Recruitment continued for consecutive days until the required sample size per institution was reached. To be included, study participants had to be 18 years or older, enrolled as undergraduate students at the respective institutions, able to read and understand English, and voluntarily consent to participate in the study. Participants were excluded from the study if they were unable to give consent due to psychological conditions or intoxication.

### Sample size calculation

The sample size was based on estimates from a similar survey conducted to estimate the utilisation of on-campus MH services among Nigerian university students. The Nigerian study yielded a 36% (p_0_ = 0.36) utilisation prevalence [30]. Assuming a comparable estimate of 31% in Zimbabwean students (p_1_ = .31), a minimum of 1066 students were required at a 95% significance level (α = 0.05) and 80% goal power (1-β). The sample size was calculated using Statistica Version 14.

### Data collection procedure

Prior to data collection, the researchers conducted a contextual analysis to determine the range of MH services available at all the institutions – see Table 1 Upon arrival, researchers approached university students in their lecture rooms and various public areas such as canteens and foyers. Researchers explained the aims and procedures to students. Participants were informed of their right to freely participate and withdraw from the study at any moment without any consequences. Volunteering students were identified, given consent forms to review, and given the opportunity to ask any questions about the study. They were then asked to sign once they had understood and agreed to participate. Once researchers obtained written informed consent, participants completed the questionnaire programmed on KoboCollect using electronic tablets. Study questionnaires were self-administered; however, researchers were nearby to offer help as needed.

**Table 1:** Available mental health services.

MH service	Institution A	Institution B	Istitution C	Institution D	Institution E
Online service	Yes	No	No	No	No
Face to face counselling	Yes	Yes	Yes	Yes	Yes
Campus psychologist	Yes	Yes	No	No	Yes
Campus chaplin	Yes	Yes	Yes	Yes	Yes
Off-campus mental health service provider, e.g., Friendship Bench	Yes	No	No	No	Yes
Mental health workshops	Yes	No	No	Yes	Yes
Mental health support groups	Yes	No	Yes	No	No
Peer educators	Yes	No	No	Yes	Yes
Mental health awareness campaigns	Yes	No	No	Yes	Yes

### Data collection instruments

#### Participants characteristics questionnaire.

Sociodemographic characteristics were elicited through an ad hoc questionnaire which captured participants’ age, sex, institution, faculty, year of study, religion, area of residence, alcohol intake, smoking status, financial adequacy and personal and familial history of MH conditions.

#### Awareness, utilisation and barriers questionnaires.

Participants were asked to indicate their knowledge of the MH services available by selecting the services provided at their respective institutions. The utilisation of MH services was assessed via a questionnaire, which identified the 12-month prevalence of MH service utilisation and the likelihood of future use. MH services preferences were gathered by asking participants about their preferred location (on-campus or off-campus), delivery mode (in-person, online, self-help, or hybrid), and delivery agent (e.g., lay counsellor, psychiatrist, psychologist). Barriers to accessing MH services were measured using questions adapted from studies that aimed to determine the barriers to MH services in other populations. This questionnaire was primarily adapted from the Barriers to Mental Health Services Scale (BMHSS) [35]. The adapted version of the questionnaire had an internal consistency of α = .788 The complete questionnaire was piloted among 35 participants to test its face validity, clarity, acceptability (respondent burden), and appropriateness. Changes were made to the questionnaire, such as omitting similar items, merging questions, and rephrasing them to improve clarity and conciseness.

### Data analysis

Data were analysed using descriptive statistics to summarise the participants’ characteristics, MH utilisation, preferences, awareness and perceived barriers to utilisation of CBMHS. Bivariate and multivariate logistic regression were used, to estimate adjusted odds ratios (AOR) and 95% confidence intervals were determined to identify factors associated with awareness, utilisation, preferences, and perceived barriers to utilisation of MH services among undergraduate students. In the bivariate analysis, crude odds ratios (CORs) with 95% confidence intervals (CIs) were estimated. Variables with p-values ≤ 0.20 were considered for inclusion in the multivariable models. Afterwards, all variables were entered simultaneously into the model. Variables were retained based on statistical significance (p < 0.05) and assessment of confounding. Model fit was evaluated using the Hosmer–Lemeshow test. Multicollinearity among independent variables was evaluated using correlation matrices, with correlation coefficients ≥ 0.8 indicating potential multicollinearity. All analyses were conducted at an α level of 0.05 using SPSS (Version 29).

## Results

### Participant characteristics

The study recruited 1,070 tertiary-level students from five institutions, with a mean age of 21.7 years (SD = 2.7). Most of the participants were: female (54.3%; n = 581), in their first year of study (43.0%; n = 460), studying engineering-related courses (31.9%; n = 341), full-time students (97.6%; n = 1044), Christian (91.0%; n = 974), resided with family (43.6%; n = 466), and rated their monthly finances as adequate for them (32.2%; n = 345). Also, most participants did not smoke (91.4%; n = 978), take alcohol (72.6%; n = 777), nor use any substances (95.4%; n = 1021). The lifetime prevalence of MH conditions was 35%, with a 12-month period prevalence of 66.7%. The most common conditions reported were depression (35.0%; n = 300) and anxiety (21.8%; n = 233). Lastly, most participants had a family member (46.1%; n = 493) or a friend (49.0%; n = 524), with a history of a MH condition – See Table 2.

**Table 2:** Participant characteristics, N = 1070.

Variable	Attribute	Frequency, n (%)
Gender	Female	581 (54.3)
	Male	475 (44.4)
	Other	14 (1.3)
*Age	Mean (SD)	21.7 (2.7)
Institution	Institution A	149 (13.9)
	Institution B	106 (9.9)
	Institution C	286 (26.7)
	Institution D	120 (11.2)
	Institution E	409 (38.2)
Academic year	First	460 (43.0)
	Second	261(24.4)
	Third	70 (6.5)
	Fourth	279 (26.1)
Faculty or course	Technical programs	41 (3.9)
	Arts And Humanities	212 (19.8)
	Health And Sciences.	325 (30.3)
	Commercials	151 (14.1)
	Engineering	341(31.9)
Enrolment classification	Full-time student	1044 (97.6)
	Part-time student	26 (2.4)
Religion	African Traditional Religion	29 (2.7)
	Christian	974 (91.0)
	Islam	14 (1.3)
	None/Atheist	22 (2.1)
	Other	31(2.9)
Residence	On-campus accommodation	212 (19.8)
	Off-campus housing	358 (33.5)
	Stays with family	466 (43.6)
	Other	34 (3.2)
Perceived financial adequacy	Very inadequate	183 (17.1)
	Inadequate	241 (22.5)
	Somewhat Inadequate	245 (22.9)
	Adequate	345 (32.2)
	Very adequate	56 (5.2)
Alcohol Intake	No	781781 (73.0)
	Yes	289289 (27.0)
Smoking status	No	978 (91.4)
	Yes	92 (8.6)
Drug and substance use	No	1021 (95.4)
	Yes	49 (4.6)
Ever experienced a mental health Condition	No	695 (65.0)
	Yes	375 (35.0)
Experienced a mental health condition in the past twelve months	No	121 (32.3)
	Yes	254 (66.7)
Type of mental health condition experienced in the last 12 months	Depression	300 (28.0)
	Anxiety	233 (21.8)
	Post-traumatic stress disorder	99(9.3)
	Bipolar disorder	34(3.2)
	Substance and drug abuse	20(1.9)
	Other	19(1.8)
Family history of mental health condition	No	577(53.9)
	Yes	493(46.1)
Having a friend with a mental health Condition	No	546(51.0)
	Yes	524(49.0)

### Awareness of campus-based mental health services

Over three-quarters of the participants (76.5%; n = 819) reported being aware of any CBMHS. Of those aware of the existing on-campus MH services, most students were aware of the existence of face-to-face counselling services (56.5%; n = 463) and peer counselling services (19.8%; n = 212). The least recognised MH services were online services/telehealth, with only 9.5% (n = 102) and 7.4% (n = 79) aware of external MH service providers (see Table 3).

**Table 3:** Awareness of on-campus mental health services.

On-campus mental health services available per institution	Attribute	Frequency, n (%)
Online service/ telehealth	No	968(90.5)
	Yes	102(9.5)
Face-to-face counselling services	No	607(56.7)
	Yes	463(43.3)
A campus-based psychologist	No	878(82.1)
	Yes	192(17.9)
Campus-based chaplain	No	889(83.1)
	Yes	181(16.9)
The university has partnered with an off-campus mental health service provider.	No	991(92.6)
	Yes	79(7.4)
Mental health workshops/talks	No	918(85.8)
	Yes	152(14.2)
Mental health support groups	No	940(87.9)
	Yes	130(12.1)
Peer educators	No	858(80.2)
	Yes	212(19.8)
Mental health awareness campaign programs	No	875(81.8)
	Yes	195(18.2)
None	No	819(76.5)
	Yes	251(23.5)

The S1 Table presents the crude odds ratios of factors associated with awareness of on-campus MH services, while Table 4 displays the adjusted analysis The Hosmer–Lemeshow goodness-of-fit test indicated good model fit (χ² = 5.247, df = 8, p = 0.731. In the multivariable analysis, only institution, experience and familial history of mental conditions were significantly associated with awareness of CBMHS. Compared to students from D, students at institution E were 50.8% less likely to be aware of the available MH services (AOR = 0.492 (95%:0.292; 0.830), p = .008). Also, participants who experienced a MH condition were 28.9% less likely to be aware of available MH services than those who had not experienced a MH disorder (AOR = 0.711 (95%:0.522; 0.968), p = .030). Last, participants with a familial history of MH conditions were almost 1.4 times more likely to be aware of available MH services than those without a familial history of MH conditions (AOR = 1.352 (95%: 1.050; 1.829), p = .05). The model explained a small proportion of the variance in mental health services awareness (Cox & Snell R^2^ = 0.029; Nagelkerke R^2^ = 0.043; −2 log likelihood = 1134.492).

**Table 4:** Factors associated with awareness of campus-based mental health services.

			95% Confidence Interval		p-value
Variable	Attribute	AOR	Lower limit	Upper limit	
Institution	Institution A	1.227	0.634	2.373	0.543
	Institution B	1.243	0.599	2.578	0.560
	Institution C	0.600	0.347	1.037	0.067
	Institution E	0.492	0.292	0.830	0.008*
	Institution D	Ref			
Experienced mental health Condition	Yes	0.711	0.522	0.968	0.030*
	No	Ref			
Family history of mental health Condition	Yes	1.352	1.050	1.829	0.050*
	No	Ref			

*Denotes statistically significant values.

### On-campus mental health service utilisation

Only a few students (16.5%; n = 177) had utilised CBMHS since they were enrolled at their institutions; of these, more than half (55.4%; n = 98) reported utilisation in the past 12 months. Out of the students who had utilised MH services in the past 12 months, most (6.7%; n = 72) reported that they had rarely used them. Most respondents were likely to recommend the on-campus MH services to others (10.8%; n = 116). Lastly, most students felt they were likely to utilise MH services in the future (54.0%; n = 578). See Table 5.

**Table 5:** Frequencies of utilisation of on-campus mental health services, N = 1070.

Variable	Attribute	Frequency, n (%)
Utilisation since enrolment	No	893 (83.5)
	Yes	177 (16.5)
Utilisation of on-campus mental health services in the past 12 months	No	79 (44.6)
	Yes	98 (55.4)
Frequency of utilisation of on-campus mental health services in the last year	Only once	34 (3.2)
	Rarely (yearly)	72 (6.7)
	Occasionally (month)	58 (5.4)
	Regularly(week)	13 (1.2)
Likelihood of recommendation of on-campus mental services to others	Very unlikely	25 (2.3)
	Unlikely	14 (1.3)
	Indifferent	22 (2.1)
	Likely	58 (5.4)
	Very likely	58 (5.4)
Likelihood of future usage of on-campus mental Services	Very unlikely	249 (23.3)
	Unlikely	243 (22.7)
	Somewhat likely	226 (21.1)
	Likely	241(22.5)
	Very likely	111(10.4)

The **S2 Table** presents the crude odds ratios of factors associated with utilising on-campus MH services, while Table 6 displays the adjusted analysis The Hosmer–Lemeshow goodness-of-fit test indicated good model fit (χ² = 3.650, df = 8, p = 0.887. In the multivariable analysis, institution, availability of MH services, preferred MH professional, and drug and substance use remained significantly associated with MH service utilisation. Students from institutions A, B, C and E were less likely to utilise CBMHS compared to students from institution D, with reductions in odds of 79.4% [AOR = .206 (95% CI: 0.125; 0.337), p < .001], 82.4% [AOR = .176 (95% CI: 0.121; 0.256), p < .001], 81.3% [AOR = .187 (95% CI: 0.131; 0.268), p < .001], and 81.1% [AOR = .199 (95% CI: 0.114; 0.348), p < .001], respectively. Further, students with a history of drug and substance use were 2.4 times more likely to utilise MH services compared to those without [AOR = 2.388 (95% CI: 1.227; 4.644), p < .001]. Next, students with access to support groups, MH service providers, psychologists, or workshops on their campus were significantly more likely to utilise MH services compared to those without, with increased odds of 2.4 times [AOR = 2.069 (95% CI: 1.26; 3.395), p = .004],1.8 times [AOR = 1.838 (95% CI: 1.009;3.348), p = .047], 1.7 times [AOR = 1.686 (95% CI:1.098; 2.59), p = .017] and 1.7 times [AOR = 1.615 (95% CI: 1.003; 2.6), p = .049] respectively. Last, participants who preferred psychologists or psychiatrists as their MH service providers were significantly less likely to utilise MH services, with reduction in odds of 47.4% [AOR = 0.526 (95% CI: 0.384; 0.72), p < .001] and 41.2% [AOR = 0.588: (95%CI: 0.358; 0.966), p = 0.036], respectively - See Table 6. The model explained a small proportion of the variance in mental health services awareness (Cox & Snell R^2^ = 0.054; Nagelkerke R^2^ = 0.091; −2 log likelihood = 900.29).

**Table 6:** Factors associated with the utilisation of campus-based mental health services.

Variable	Attribute	Adjusted	95% Confidence interval		p-value
		Odds ratio	Lower limit	Upper limit	
Institution	Institution A	0.206	0.125	0.337	<.001*
	Institution B	0.199	0.114	0.348	<.001*
	Institution C	0.176	0.121	0.256	<.001*
	Institution E	0.187	0.131	0.268	<.001*
	Institution D	Ref			
Drug and substance use	Yes	2.388	1.227	4.644	< 0.001*
	No	Ref			
Available mental health services:					
Psychologist	Yes	1.686	1.098	2.59	0.017*
	No	Ref			
External mental health service provider	Yes	1.838	1.009	3.348	0.047*
	No	Ref			
Workshops	Yes	1.615	1.003	2.6	0.049*
	No	Ref			
Support groups	Yes	2.069	1.26	3.395	0.004*
	No	Ref			
Preferred professional:					
Psychologist	Yes	0.526	0.384	0.72	<.001*
	No	Ref			
Psychiatrist	Yes	0.588	0.358	0.966	0.036*
	No	Ref			

*Denotes statistically significant values.

### Perceived barriers to utilisation

The most endorsed barriers to accessing CBMHS included: fear of being seen as weak for having a MH condition (46.7%, n = 499), feelings of shame or embarrassment (44.2%, n = 472), past bad experiences with CBMHS (67.9, n = 727), the belief that professional help would likely be ineffective (61.3%, n = 656), being too unwell to ask for help (56%, n = 599), and concerns about cost (42.7%, n = 457) – See S4 Table

The S3 Table presents the crude odds ratios of factors associated with barriers to accessing on-campus MH services, while Table 8 displays the adjusted analysis. The Hosmer–Lemeshow goodness-of-fit test indicated good model fit (χ² = 13.427, df = 8, p = 0.098. In the multivariable analysis, only institution, experience with MH condition(s), availability of certain on-campus MH services, and lifetime utilisation of on-campus MH services were associated with barriers to service utilisation. First, students at institutions with an MH service provider were twice as likely to perceive barriers to utilising CBMHS compared to students at an institution without an MH service provider (AOR = 2.016 [95% CI: 1.157; 3.514], p = .013). Second, students who had participated in MH service workshops were 1.6 times more likely to perceive barriers to utilisation of MH services compared to those who did not participate in CBMHS workshops [AOR = 1.615 (95% CI: 1.089; 2.395), p = .017]. Third, participants who had experience of a MH condition in their lifetime were 1.5 times more likely to perceive barriers to CBMHS than students who had never experienced a MH condition in their lifetime [AOR = 1.533 (95% CI:1.164; 2.019), p = .002]. Fourth, participants with a friend diagnosed with a MH condition were 1.4 times more likely to face barriers to utilisation of CBMHS than those who had no friend diagnosed with a MH condition [AOR = 1.437 (95% CI: 1.109; 1.863), p = .006]. Fifth, compared to students at D, students at institution C are 53.6% more protected from experiencing the barriers to accessing MH services [AOR = 0.464 (95% CI:0.295; 0.728), p < .001] while students at B are 62% more protected from experiencing barriers to accessing MH services [AOR = 0.380 (95% CI: 0.219; 0.661), p < .001] and students at institution A are 43.5% more protected from experiencing barriers to accessing MH services [AOR = 0.565 (95% CI: 0.335; 0.954), p = .033]. Lastly, students who had utilised CBMHS since enrolment were 40.9% less likely to perceive barriers to utilisation of CBMHS compared to those who had never utilised CBMHS, [AOR = 0.591 (95% CI: 0.418; 0.835), p = 0.008]. The model explained a small proportion of the variance in mental health services awareness (Cox & Snell R^2^ = 0.086; Nagelkerke R^2^ = 0.064; −2 log likelihood = 1398.32) (Table 7).

**Table 7:** Factors associated with barriers to the utilisation of campus-based mental health services.

Variable	Attribute	Odds ratio	95% Confidence interval		p-value
			Lower limit	Upper limit	
Institution	Institution A	.565	0.335	0.954	.033*
	Institution B	.380	0.219	0.661	<.001*
	Institution C	.464	0.295	.728	<.001*
	Institution E	.706	0.455	1.095	.120
Institution	Institution D	Ref			
Ever experienced a mental health condition*	Yes	1.533	1.164	2.019	.002*
	No	Ref			
Friend diagnosed with mental health disorder*	Yes	1.437	1.109	1.863	.006*
	No	Ref			
Available services:					
Face –to- face*	Yes	1.392	1.071	1.809	.013*
	No	Ref			
On-campus MHS provider availability, e.g., Friendship bench*	Yes	2.016	1.157	3.514	.013*
	No	Ref			
Workshops*	Yes	1.615	1.089	2.395	.017*
	No	Ref			
Utilisation since enrolment*	Yes	.591	.418	0.835	.003*
	No	Ref			

*Denotes statistically significant value.

**Table 8:** Students’ preferences for mental health services.

Preference Category	Variable	Attribute	Frequency, n (%)
Location and intervention delivery Format	Delivery mode	Individual therapy on campus	563 (52.6)
		Individual therapy off-campus	356 (33.3)
		Group therapy on campus	272 (25.4)
	Location and mode	In-person campus-based	603 (65.4)
		In-person off-campus	483 (45.1)
		Digital	440 (41.1)
Preferred mental health professional	Psychologist	No	479 (44.8)
		Yes	591 (55.2)
	Psychiatrist	No	895 (83.6)
		Yes	175 (16.4)
	Counsellor	No	583 (54.5)
		Yes	487 (45.5)
	Peer counsellor	No	757 (70.7)
		Yes	313 (29.3)
	Dean of Students	No	970 (90.7)
		Yes	100 (9.3)
	Janitor	No	1019 (95.2)
		Yes	51(4.8)
	Social worker	No	538 (50.3)
		Yes	532 (49.7)
	Self-help service	No	185 (17.3)
		Yes	885 (82.7)

### Preferences

Most students (52.6%; n = 563) indicated a preference for individual on-campus therapy. Two-thirds of the students preferred in-person, campus-based services (65.4%; n = 603). Lastly, more than three-quarters of the students (82.7%; n = 885) chose self-help as a viable source of MH support during a crisis, and psychologists were the leading preferred MH service providers (55.2%, n = 591) - See Table 8.

## Discussion

This study examined the awareness and utilisation of CBMHS among Zimbabwean tertiary students, including the barriers, facilitators, and preferences that influence their access to support. 76.5% of the participants were aware of any CBMHS, yet only 16.5% of students utilised these. Awareness and use were linked to personal or family MH condition history, past experiences with on-campus services, support availability, and institutional factors.

### Awareness of campus-based mental health services and associated factors

In this study, results are consistent with previous research, indicating that a significant proportion of students are aware of available MH resources [36,37]. A scoping review yielded awareness rates in the 13–54% range [38]. Similarly, a cross-sectional study conducted in the United States (N = 292) revealed that two-thirds of the tertiary students were aware of the MH services available on campus [39]. In the United States study, although students were informed about existing services during first-year orientation, the campaign messages lacked crucial details about the type and location of services, instead providing only general information about their existence [39]. Regionally, similar discrepancies can be observed between our study and a cross-sectional study of 1,424 students at Pwani University in Kenya, which reported only 29% of students were aware of the MH services available at university [40]. In this study, discrepancies were found in awareness of CBMHS across the five universities recruited. This difference could stem from variable institutional policies and infrastructure, including health promotional efforts. For instance, universities with higher levels of engagement in promoting MH services often use targeted campaigns, workshops, or partnerships with MH organisations, which can significantly boost awareness levels [37]. Also, the accessibility and visibility of these services on campus could influence awareness levels. For example, clear signage, centralised help centres, or integration of MH resources into student wellness services might make services better known across campuses [41–43]. Cultural stigma around MH can prevent students at some institutions from seeking or discussing support services [43]. Therefore, universities can enhance student support by implementing campus MH policies, improving resource accessibility, fostering partnerships, raising awareness, reducing stigma, and regularly monitoring and revising the policies to ensure their effectiveness and support student well-being and academic success [17]. Also, in this study, participants with a familial history of MH disorders were more likely to be aware of available MH services. These results are consistent with an Egyptian cross-sectional study (N = 1740) among undergraduates, which found that participants with a familial history of MH issues were 1.8 times more likely to be aware of MH services than those without a familial history of MH conditions [44]. Exposure to mental health conditions in families increases awareness of services and treatment options through open discussions and direct experiences [45]. MH awareness and knowledge are closely linked to personal experience [45], aligning with social learning theory, which asserts that observation influences behaviour [46].

Furthermore, participants who experienced a MH condition were 28.9% less likely to be aware of available CBMHS than those who had not experienced a MH condition. This suggests that individuals who have experienced a MH condition may be receiving services elsewhere (e.g., public hospitals, private MH care), resulting in them not seeking or considering campus-based services. This appears unconventional yet can be explained by the reliance of students with lived experience of MH conditions on other forms of psychological support. This includes social support networks such as colleagues, mentors, friends and family [47]. This accentuates their preference of social support over institutional services thus highlighting a lack of trust due to concerns of confidentiality, fear of being judged and their perceived effectiveness [48]. Additionally, barriers such as a lack of perceived need for professional help, and internalised stigma reduce the students’ need to seek help from institutional services thus lowering the awareness of available services [49]. Also trust in institutional services is pivotal to awareness of MH services, with distrust associated with decreased awareness [15]. Alternatively, students with experience of MH condition may be focusing on managing their condition rather than actively seeking information about available services. They might perceive stress or anxiety related to academics as a normal aspect of college life, resulting in the normalisation of distress among students, and this has been observed in other studies [43]. However, these results diverge from past studies, for example, an American cross-sectional study (N = 266) found that students who had experienced MH conditions were more likely to be knowledgeable of CBMHS (*B* = .145, *t* = 2.440, *p* = .015) [50]. The US study suggests that personal experience enhances the knowledge of existing support services [50]. The experiential learning theory suggests that awareness of CBMHS can be shaped by direct experience and reflections on MH services and campaigns, ultimately influencing their knowledge and understanding of MH care [51]. Elsewhere, Bangladeshi university students (N = 2036) with good MH were less likely to have higher awareness of MH services than those with poor MH (OR 0.72, 95%CI:.60–.87, p < .01) [52]. Further studies are needed to understand the link between awareness of existing on-campus MH services and the experience of a MH condition. Nevertheless, the variable level of awareness of existing on-campus MH services across the study sites underscores the critical need for universities to prioritise continuous, universal/campus-wide health promotional initiatives, including increasing coverage of MH services to increase awareness, as this is a critical step towards reducing the burden of disease through preventative and health promotive approaches [44,25].

### Utilisation of campus-based mental health services and associated factors

Our study reflects low utilisation of CBMHS. A systematic review and meta-analysis of undergraduate students from 10 European studies showed a utilisation prevalence range from 13.1% to 68.1% [53]; our study aligns with the lower range. Utilisation patterns observed in our study are also consistent with evidence from other LMICs, where engagement with campus-based and formal mental health services remains low despite substantial need. Studies from Ethiopia, Nigeria and South Africa show comparable utilisation ranges and highlight similar determinants, including limited awareness, preference mismatches, structural barriers and stigma. For instance, in the Ethiopian cross-sectional study of 3,240 undergraduate students, only 28.7% utilised MH services, despite a high prevalence of mental distress in this setting [45]. Low utilisation in the Ethiopian study was attributed to a lack of awareness and perceived barriers to utilisation [45]. Similarly, a Nigerian study of 450 undergraduate students reported a lifetime prevalence of MH service use of 36%, with cost concerns and stigma associated with seeking help driving he low utilisation [30]. In the Nigerian study, about 60% of participants rarely used MH services, and 46% were unlikely to use them in the future [30]. Further a Ugandan cross-sectional study on the knowledge, attitude and perceptions of undergraduate medical students (N = 259) reported negative perceptions may potentially hinder utilisation of MH services [54]. Suboptimal uptake and reluctance to use existing services are areas of concern. These issues may stem from low awareness, preference mismatch, previous dissatisfaction, personal characteristics, and fear of stigma. Consequently, individual students may turn to alternative support systems, such as peers, family, and self-management [43,55,56].

Low utilisation is multifaceted and is driven by various factors. In this study, discrepancies were found in the utilisation of CBMHS among the recruited universities. Some students at the recruited universities were more aware of MH services, increasing their likelihood of seeking help. Secondly, variations in institutional culture, policies, or student characteristics may influence MH service use [25,53]. For example, participants who preferred psychologists or psychiatrists as their service providers were 47.4% and 41.2% respectively less likely to utilise CBMHS. Students may avoid seeking professional help for mental health concerns due to believing their issues aren’t serious enough, preferring self-management, getting help elsewhere, lacking information about available resources, or not having access to their preferred services on campus [49]. Also, the shortage of these specialist services within our context may explain the observed association, as the unavailability and inaccessibility are associated with long waiting times and resultant lower utilisation. However, in a Nigerian cross-sectional study (N = 450), participants who preferred psychiatrists as their service providers were more likely to utilise CBMHS [30]. Psychiatrists were viewed as specialised experts providing a comprehensive assessment and treatment of mental disorders, which can instil confidence in students that they can receive thorough care, hence increasing their likelihood to seek help [30]. Our study also observed that students with a history of drug and substance abuse were 2.4 times more likely to utilise campus-based MH services. This suggests that prior experiences with substance use may lead to a greater need for MH support, given the multiple impacts of substance use on MH [57]. Similarly, a cross survey of South African university students (N = 1402) found that students with a history of drug and substance use were almost three times more likely to utilise MH services compared to those without [AOR = 2.89 (95%: 1.11; 7.50) p < .05] [58].

### Perceived barriers to the utilisation of campus-based mental health services and associated factors

In this study, students experienced several barriers to accessing CBMHS, with stigma-related barriers, particularly the perspectives of family or friends concerning MH and distress disclosure. Most of the participants (67.9%) cited previous bad experiences with CBMHS as an intrusive barrier. Negative experiences can undermine trust, diminishing confidence in the services provided. Additionally, students may be reluctant to seek help again due to the fear that they might encounter a similar negative experience in the future [59]. Notably, 61.3% of participants also expressed reluctance to seek professional help, believing that professional care would not help. This may be attributed to a lack of awareness of MH care and treatment options [60]. Additionally, students may feel uncomfortable sharing their distress with professionals as compared to family and friends [30,56]. Furthermore, being too unwell to ask for help was a significant barrier, with almost half of the participants highlighting this issue. This may be because students suffering from CMDs may experience physical symptoms, such as fatigue, low energy, and irritability, which have a broad impact on their quality of life and motivation to seek help [49].

In this study, students with a family member or friend with a MH condition are more likely to perceive barriers to using CBMHS, often due to negative experiences shared by their loved ones. While this familiarity can heighten awareness and potentially help overcome barriers such as stigma and lack of knowledge, it may also lead to increased reluctance to seek help, stemming from fears of facing similar challenges [30,61,62]. Therefore, it is crucial to provide targeted education and students with second-hand lived experience of MH condition(s) in MH treatment efforts to reduce stigma and attitude-related barriers. Also, students who attended mental health workshops were more likely to face barriers to the utilisation of CBMHS. This finding is unexpected because workshops typically aim to inform students about available MH services, including where and how to access them, reduce stigma associated with seeking help, and provide a supportive environment that enhances uptake of existing services [63]. Conversely, workshops provide students with a comprehensive understanding of the challenges encountered by individuals who use MH services, which may inadvertently deter them from seeking care [23]. Additionally, ineffective workshop delivery and consequent dissatisfaction with the provided information may lead to a decline in the utilisation of existing campus services [23]. Nevertheless, further studies, including qualitative inquiry, are required to understand better the relationship between workshop attendance and the barriers to utilising campus-based mental health services.

We also observed that students who utilised CBMHS since enrolment were less likely to face barriers. Utilisation of services can increase familiarity with available CBMHS and what they entail, enhancing service uptake [59]. Engaging with MH services early allows students to recognise and manage issues before they escalate, which may result in better outcomes and fewer obstacles to future use of available support services [44,53,49]. Significantly, the accessibility and convenience of CBMHS can minimise perceived access barriers, particularly compared to off-campus alternatives, which may encourage future use [64]. Also, students at institutions with resident partner MH service providers, such as Friendship Bench, perceived more barriers to utilisation than those without. This was unexpected, as other studies have shown that onsite external partners facilitate early access to services, including destigmatising mental healthcare access [53,19]. However, students may find it challenging to navigate the system, particularly if they are hesitant to disclose their distress or lack awareness of available support, which can result in lower utilisation of services [43]. Also, a limited understanding of the partnership’s role, benefits or services might contribute to perceived barriers, and students may also have different expectations about what MH services should entail [30,25]. Additionally, our study suggests that a lack of awareness about the services and their benefits might contribute to this perception, as 61.3% of participants believed professional help would not be effective. This finding highlights the importance of not only providing MH services but also ensuring they meet students’ needs and expectations. Tertiary institutions should proactively address barriers to MH service utilisation by implementing awareness campaigns that educate students on early identification of MH symptoms and available campus-based services [37]. However, the effectiveness of such awareness initiatives is contingent on governance frameworks that guide CBMHS delivery, including institutional policies and dedicated funding for CBMHS initiatives which were largely absent in this study’s context. Systematic reviews reveal a scarcity of accessible policy frameworks supporting CBMHS across Sub-Saharan Africa (SSA) [17]. In the Zimbabwean context, no formal university MH policies were identified in the sampled universities. This contrasts with settings such as Australia, where structured university MH frameworks have been associated with improved awareness, access, and utilisation of campus MH services [65]. Further, these gaps also reflect broader financing patterns that prioritise campus SRH and infectious diseases over MH, thereby reinforcing fragmented and under-resourced responses within higher education settings. The institutional funding landscape and priorities reflect similar national and global priorities. Additionally, institutions must invest in service evaluations to ensure their offerings meet students’ needs, ultimately creating a supportive environment and encouraging students to seek help when necessary [43,63].

### Preferences of campus-based mental health services

In our study, participants showed a greater acceptance of in-person therapy conducted on campus, followed by digital therapy, with blended therapy (a combination of physical and online sessions) being the least preferred. This finding aligns with the results from a multi-centre cross-sectional study of German university students (N = 1,376), which revealed a preference for in-person MH services. Specifically, in the German study 76% of the students preferred off-campus and 71% preferred on-campus services, as opposed to digital therapy (19%) and blended therapy (9%) [66]. Similarly, a Mexican cross-sectional study of first-year students (N = 7849) found that 38% of students preferred in-person therapy over other services [67]. Students may prefer in-person on-campus services because they offer a convenient, accessible, and supportive environment that fosters face-to-face interaction, greater therapeutic alliance, immediate support and a sense of community and security [21,68]. Also, most students preferred individual therapy over group therapy (85.9% vs. 59.7%). These results are comparable to those of a German cross-sectional cohort study (N = 1,376), which found that 76% preferred individual therapy and 67% opted for group therapy [66]. The German study found that preferences for individual therapy were due to embarrassment, stigma, and beliefs about effectiveness: reasons echoed in our study. Most participants (82.7%) in the study showed a strong preference for self-help services, despite their absence across five tertiary institutions. This highlights a growing inclination towards autonomous MH care [56]. Self-help services, lauded for their convenience and alignment with high literacy rates among tertiary students, present an accessible opportunity to bridge the care gap, especially in an increasingly digitalised world [56]. Also, more students opted for peer counsellors as their MH service provider of choice over professionals (e.g., psychiatrists). This is comparable to a US study (N = 14,175) in which counselling services were preferred from non-professionals, such as friends (70.5%) [41]. The preference for peer counsellors might be attributed to their relatability, approachability, and shared experiences, which can offer comfort and understanding in a non-clinical setting [69]. However, this may be accompanied by confidentiality concerns thus potentially further limiting the utility of services and limiting participation despite being preferred [13]. Peer support groups provide a task sharing model that is potentially cost effective and culturally acceptable among university students [70]. This harnesses the resource limitations Given the recommendation by the WHO to task-shift care, tertiary institutions should prioritise equipping peer counsellors with the necessary knowledge to help students increase utilisation [69].

### Study strengths and limitations

To our knowledge, this study is one of the few that have examined the awareness, perceived utility, and barriers to accessing on-campus mental health services in Sub-Saharan Africa. Our study offers valuable insights into improving the utilisation and reach of mental health services in tertiary institutions. Also, recruiting a large sample across five tertiary institutions increases the study’s internal and external validity. However, the application of convenience sampling could have resulted in recruitment or selection bias, for instance, overrepresentation of more accessible or engaged students. Also, the study’s metropolitan context may limit the transferability of findings to rural or less-resourced institutions. Ideally, participants would have been randomly selected from more universities across the country; however, this was not feasible due to resource limitations. The collection tools employed were not formally validated within our context, which may have led to measurement and misclassification biases. Future studies should address these limitations by validating the tools formally to ensure reliability and accuracy before conducting research. Importantly, the use of a quantitative design without qualitative methods limits a fuller understanding of the utilisation, preferences, and barriers to accessing CBMHS. Future mixed-methods research that triangulate quantitative and qualitative findings is warranted to help capture the nuances and unique experiences across participants that may be missed by standardised outcome measures. Last, future studies should consider institutional perspectives and expert informant interviews to unpack structural barriers and policy frameworks to inform the development of bespoke policies and interventions.

## Conclusion

Our study revealed a high level of awareness and low utilisation of CBMHS among tertiary-level students. The main barriers to using these services were related to attitudes and a lack of information. Tertiary institutions should promote MH awareness through tailored, campus-wide health initiatives that direct students to the available MH services such as utilising digital platforms and on-campus communication channels such as radio stations for dissemination of information. Institutions must consider the introduction of mandatory MHL courses for all students to increase awareness and improve help-seeking behaviours. CBMHS must be aligned with student preferences, and there should be ongoing assessments of their acceptability to foster a supportive environment for students’ well-being. Tertiary institutions must incorporate decentralisation of MH services by adopting interventions such as the Friendship Bench –a task shifting model, at their respective campuses. Further, the training of university students to provide MH support and collaboration with organisations that provide youth counselling services increases their preferred peer-to-peer support. Lastly, there is a need to leverage digital technologies to offer self-help MH services and bridge the care gap within universities. - See S5 Table

## Supporting information

S1 Table Factors associated with awareness, unadjusted odds ratios. (DOCX)

S2 Table Factors associated with utilisation unadjusted odds ratios. (DOCX)

S3 Table Factors associated with barriers to utilisation. (DOCX)

S4 Table Barriers to access of MHS. (DOCX)

S5 Table Recommendations. (DOCX)

S1 Data Data. (XLSX)
