# Supplementary material for: Awareness, utility and preferences of campus-based mental health services at tertiary institutions in Harare, Zimbabwe: A cross-sectional study
Source: PLOS Glob Public Health. 2026 May 6;6(5):e0005107. doi: 10.1371/journal.pgph.0005107 (PMC13148715; doi:10.1371/journal.pgph.0005107)
Supplement: S1 Table — (DOCX) [file pgph.0005107.s001.docx]

# **Supporting information**

## **S1 Table: Factors associated with awareness of mental health services: Unadjusted odds ratios**

|  |  | **Crude** | **95% Confidence Interval** | |  |
| --- | --- | --- | --- | --- | --- |
| **Variable** | **Attribute** | **Odds Ratio** | **Lower**  **limit** | **Upper**  **limit** | **p-value** |
| Institution | Institution D | .777 | .378 | 1.598 | .493 |
|  | Institution A | .952 | .468 | 1.934 | .891 |
|  | Institution C | .490 | .267 | .900 | **.021** |
|  | Institution B | .392 | .218 | .705 | **.002** |
|  | institution E | Ref | . | . |  |
| Gender | Female | .957 | .720 | 1.273 | .764 |
|  | Male | Ref |  |  |  |
| Age |  | 1.003 | .951 | 1.057 | .924 |
| Year of study | First | 1.447 | 1.029 | 2.036 | .034 |
|  | Second | 1.616 | 1.084 | 2.409 | .019 |
|  | third | 1.079 | .600 | 1.941 | .799 |
|  | Fourth | 1.447 | 1.029 | 2.036 | .034 |
| Religious beliefs | ATR | 1.093 | .339 | 3.525 | .881 |
|  | Christianity | 1.158 | .511 | 2.624 | .725 |
|  | Islam | .870 | .212 | 3.566 | .846 |
|  | None /atheist | .745 | .223 | 2.487 | .633 |
|  | Other | Ref |  |  |  |
| Perceived financial adequacy | Very inadequate | .955 | .471 | 1.937 | .899 |
|  | Inadequate | .999 | .502 | 1.988 | .997 |
|  | Somewhat adequate | .997 | .501 | 1.983 | .993 |
|  | Adequate | .985 | .505 | 1.923 | .966 |
|  | Very adequate | Ref |  |  |  |
| Residential area | On-campus accommodation | .840 | .345 | 2.045 | .701 |
|  | Off-campus housing | .931 | .391 | 2.218 | .871 |
|  | Stays with family | .782 | .332 | 1.844 | .575 |
|  | Other | .840 | .345 | 2.045 | .701 |
| Alcohol intake* | No | 1.299 | .953 | 1.771 | .098 |
|  | Yes | Ref |  |  |  |
| Smoking status* | No | .837 | .495 | 1.416 | .507 |
|  | Yes | Ref |  |  |  |
| Drug and substance intake* | No | 1.323 | .700 | 2.501 | .389 |
|  | Yes | Ref |  |  |  |
| Lifetime experience of mental health | No | 1.339 | 1.001 | 1.792 | **.049** |
| condition* | Yes | Ref |  |  |  |
| Experienced with mental health issues in | No | 1.888 | 1.115 | 3.197 | **.018** |
| the past year* | Yes | Ref |  |  |  |
| History of a family member being | No | .816 | .613 | 1.086 | .163 |
| diagnosed with a mental condition * | Yes | Ref |  |  |  |
| History of a friend being diagnosed with a | No | .848 | .638 | 1.126 | .253 |
| mental condition * | Yes | Ref |  |  |  |
| Enrolment type* | Full-time student | .587 | .200 | 1.719 | .331 |
|  | Part-time student | Ref |  |  |  |
| Experienced barriers in accessing on- | No | 1.319 | .988 | 1.759 | .060 |
| campus mental health services* | Yes | Ref |  |  |  |
| Utilised on-campus mental health services | No | .093 | .041 | .212 | **<.001** |
| since enrolment* | Yes | Ref |  |  |  |
| Utilised on-campus mental health services | No | .391 | .070 | 2.190 | .285 |
| in the past year* | Yes | Ref |  |  |  |
| Recommend on-campus mental | Very unlikely |  |  |  |  |
| health services to others | Unlikely | .228 | .013 | 3.890 | .307 |
|  | Somewhat likely | .175 | .015 | 2.041 | .164 |
|  | Likely | .491 | .043 | 5.572 | .566 |
|  | Very likely |  |  |  |  |
| Would consider utilising self-help mental | No | 1.209 | .794 | 1.842 | .377 |
| health services* | Maybe | .815 | .599 | 1.108 | .192 |
|  | Yes | Ref |  |  |  |
