# Supplementary material for: Awareness, utility and preferences of campus-based mental health services at tertiary institutions in Harare, Zimbabwe: A cross-sectional study
Source: PLOS Glob Public Health. 2026 May 6;6(5):e0005107. doi: 10.1371/journal.pgph.0005107 (PMC13148715; doi:10.1371/journal.pgph.0005107)
Supplement: S2 Table — (DOCX) [file pgph.0005107.s002.docx]

## **S2 Table: Factors associated with the utilisation of on-campus mental services – unadjusted/crude odds ratios**

| **Variable** | **Attribute** | **Crude** | **95% Confidence Interval** | | **p-value** |
| --- | --- | --- | --- | --- | --- |
|  |  | **Odds Ratio** | **Lower**  **limit** | **Upper limit** |  |
| Institution | Institution D | 1.793 | .911 | 3.525 | .091 |
|  | Institution A | 1.478 | .762 | 2.867 | .248 |
|  | Institution C | .836 | .443 | 1.576 | .579 |
|  | Institution E | 1.043 | .576 | 1.892 | .888 |
|  | Institution B | Ref | . | . | . |
| Gender | Female | 1.238 | .892 | 1.718 | .201 |
|  | Male | Ref |  |  |  |
| Year of study | First | .658 | .437 | .990 | .044 |
|  | Second | 1.086 | .706 | 1.671 | .708 |
|  | Third | 1.325 | .702 | 2.500 | .386 |
|  | Fourth | Ref |  |  |  |
| Religious beliefs | ATR | 1.587 | .475 | 5.307 | .453 |
|  | Christianity | .795 | .321 | 1.969 | .619 |
|  | Islam | 1.136 | .240 | 5.390 | .872 |
|  | None/ atheist | .926 | .228 | 3.765 | .914 |
|  | Other | Ref |  |  |  |
| Financial status | Very inadequate | 1.224 | .527 | 2.841 | .639 |
|  | Inadequate | 1.088 | .476 | 2.487 | .841 |
|  | Somewhat inadequate | 1.101 | .483 | 2.512 | .818 |
|  | Adequate | 1.340 | .604 | 2.973 | .471 |
|  | Very adequate | Ref |  |  |  |
| Residential area | On-campus accommodation | .816 | .330 | 2.013 | .658 |
|  | Off -campus housing | .746 | .310 | 1.793 | .512 |
|  | Stays with family | .740 | .311 | 1.761 | .496 |
|  | Other | Ref |  |  |  |
| Alcohol* | No | 1.143 | .788 | 1.657 | .481 |
|  | Yes | Ref |  |  |  |
| Smoking* | No | .561 | .339 | .927 | .024 |
|  | Yes | Ref |  |  |  |
| Drug and substance use* | No | .386 | .208 | .718 | .003 |
|  | Yes | Ref |  |  |  |
| Ever experienced a mental health condition* | No | .728 | .523 | 1.012 | .059 |
|  | Yes | Ref |  |  |  |
| Family history of mental condition* | No | .936 | .677 | 1.292 | .686 |
|  | Yes | Ref |  |  |  |
| Friend diagnosed of a mental condition* | No | .914 | .662 | 1.262 | .585 |
|  | Yes | Ref |  |  |  |
| Enrolment type | Full-time student | .528 | .219 | 1.275 | .156 |
|  | Part-time student | Ref |  |  |  |
| Available services: |  |  |  |  |  |
| Online* | No | .481 | .301 | .768 | .002 |
|  | Yes | Ref |  |  |  |
| Face-to-face* | No | .485 | .350 | .673 | <.001 |
|  | Yes | Ref |  |  |  |
| Psychologist* | No | .517 | .355 | .753 | <.001 |
|  | Yes | Ref |  |  |  |
| Chaplain* | No | .617 | .416 | .914 | .016 |
|  | Yes | Ref |  |  |  |
| MHS provider* | No | .366 | .222 | .604 | <.001 |
|  | Yes | Ref |  |  |  |
| Workshops* | No | .384 | .259 | .568 | <.001 |
|  | Yes | Ref |  |  |  |
| Support groups* | No | .322 | .214 | .484 | <.001 |
|  | Yes | Ref |  |  |  |
| Peer educators* | No | .525 | .364 | .757 | <.001 |
|  | Yes | Ref |  |  |  |
| Awareness campaigns* | No | .458 | .317 | .663 | <.001 |
|  | Yes | Ref |  |  |  |
| Self –help* | No | .722 | .453 | 1.150 | .170 |
|  | Maybe | .700 | .488 | 1.004 | .053 |
|  | Yes | Ref |  |  |  |
| Professional preference: |  |  |  |  |  |
| Psychologist* | No | 1.202 | .870 | 1.661 | .263 |
|  | Yes | Ref |  |  |  |
| Psychiatrist* | No | 1.373 | .857 | 2.201 | .187 |
|  | Yes | Ref |  |  |  |
| Counsellor* | No | 1.231 | .888 | 1.707 | .212 |
|  | Yes | Ref |  |  |  |
| Social worker* | No | .950 | .663 | 1.360 | .777 |
|  | Yes | Ref |  |  |  |
| Peer counsellor* | No | .901 | .635 | 1.279 | .560 |
|  | Yes | Ref |  |  |  |
| Chaplain* | No | .967 | .639 | 1.464 | .874 |
|  | Yes | Ref |  |  |  |
| Dean of students* | No | .674 | .408 | 1.115 | .125 |
|  | Yes | Ref |  |  |  |
| Janitor* | No | .804 | .395 | 1.636 | .547 |
|  | Yes | Ref |  |  |  |
| Therapist* | No | 1.177 | .852 | 1.626 | .323 |
|  | Yes | Ref |  |  |  |
| Other * | No | 1.943 | .825 | 4.579 | .129 |
|  | Yes | Ref |  |  |  |
| MH services awareness* | No | .093 | .041 | .212 | <.001 |
|  | Yes | Ref |  |  |  |

* Denotes all variables for which Yes attribute was used as the reference, bolded p-values denote statistically significant values p≤0.05
