# Supplementary material for: Awareness, utility and preferences of campus-based mental health services at tertiary institutions in Harare, Zimbabwe: A cross-sectional study
Source: PLOS Glob Public Health. 2026 May 6;6(5):e0005107. doi: 10.1371/journal.pgph.0005107 (PMC13148715; doi:10.1371/journal.pgph.0005107)
Supplement: S3 Table — (DOCX) [file pgph.0005107.s003.docx]

## **S3 Table: Factors associated with barriers in the utilisation of on-campus mental services – unadjusted/crude odds ratios**

|  |  |  | **95% Confidence Interval** | |  |
| --- | --- | --- | --- | --- | --- |
| **Variable** | **Attribute** | **Odds Ratio** | **Lower limit** | **Upper limit** | **p-value** |
| Institution | Institution D | 2.164 | 1.268 | 3.692 | **.005** |
|  | Institution A | 1.562 | .946 | 2.577 | **.081** |
|  | Institution C | 1.096 | .700 | 1.714 | .689 |
|  | Institution B | 1.939 | 1.260 | 2.985 | .**003** |
|  | Institution E | Ref |  |  |  |
| Gender | Female | .361 | .100 | 1.308 | .121 |
|  | Male | .316 | .087 | 1.148 | .080 |
|  | Other | Ref |  |  |  |
| Year of study | First | .723 | .535 | .977 | **.035** |
|  | Second | 1.011 | .717 | 1.427 | .949 |
|  | Third | .808 | .477 | 1.370 | .429 |
|  | Fourth | Ref |  |  |  |
| Religious beliefs | ATR | .386 | .136 | 1.094 | .073 |
|  | Christianity | .806 | .387 | 1.678 | .564 |
|  | Islam | .351 | .095 | 1.301 | .117 |
|  | None /atheist | 1.684 | .515 | 5.505 | .388 |
|  | Other | Ref |  |  |  |
| Financial adequacy | Very inadequate | .911 | .494 | 1.679 | .765 |
|  | Inadequate | .721 | .398 | 1.304 | .279 |
|  | Somewhat adequate | .907 | .501 | 1.642 | .748 |
|  | Adequate | .757 | .425 | 1.347 | .344 |
|  | Very inadequate | Ref |  |  |  |
| Alcohol intake* | No | .879 | .668 | 1.157 | .358 |
|  | Yes | Ref |  |  |  |
| Smoking habits* | No | .684 | .439 | 1.066 | .094 |
|  | Yes | Ref |  |  |  |
| Drug and substance use* | No | 1.030 | .579 | 1.833 | .920 |
|  | Yes | Ref |  |  |  |
| Experienced mental condition* | No | .594 | .459 | .769 | **<0.001** |
|  | Yes | Ref |  |  |  |
| Experienced mental condition in the past year* | No | 1.146 | .728 | 1.806 | .556 |
|  | Yes | Ref |  |  |  |
| Mental health condition | Depression | .353 | .210 | .592 | **<0.001** |
|  | Anxiety | .958 | .620 | 1.478 | .845 |
|  | PTSD | .802 | .493 | 1.305 | .375 |
|  | Bipolar disorder | .967 | .463 | 2.021 | .928 |
|  | Substance abuse | 1.197 | .477 | 3.005 | .702 |
|  | Other | 1.311 | .514 | 3.344 | .570 |
| Family member diagnosed of a MH condition* | No | .760 | .596 | .969 | **.027** |
|  | Yes | Ref |  |  |  |
| Friend diagnosed of a MH condition* | No | .617 | .484 | .787 | **<.001** |
|  | Yes | Ref |  |  |  |
| Enrolment type | Full-time | 2.055 | ,924 | 4.571 | .077 |
|  | Part-time | Ref |  |  |  |
| **Available services:** |  |  |  |  |  |
| Psychologist* | No | .695 | .504 | .958 | **.027** |
|  | Yes | Ref |  |  |  |
| Chaplain* | No | .740 | .533 | 1.026 | .071 |
|  | Yes | Ref |  |  |  |
| MHS provider, e.g. Friendship Bench* | No | .494 | .299 | .815 | **.006** |
|  | Yes | Ref |  |  |  |
| Workshops* | No | .614 | .429 | .879 | **.008** |
|  | Yes | Ref |  |  |  |
| Support groups* | No | .792 | .545 | 1.153 | .224 |
|  | Yes | Ref |  |  |  |
| Peer educators* | No | .831 | .612 | 1.127 | .234 |
|  | Yes | Ref |  |  |  |
| Awareness campaigns* | No | .875 | .639 | 1.199 | .407 |
|  | Yes | Ref |  |  |  |
| None* | No | .758 | .568 | 1.012 | 0.60 |
| Utilisation since enrolment* | No | 1.415 | 1.025 | 1.956 | **.035** |
|  | Yes | Ref |  |  |  |
| Utilisation past year* | No | .505 | .276 | .922 | **.026** |
|  | Yes | Ref |  |  |  |
| Frequency of use in the past year | Once | .387 | .104 | 1.440 | .157 |
|  | Rarely (Yearly) | .559 | .167 | 1.874 | .346 |
|  | Occasionally (Monthly) | .718 | .210 | 2.457 | .597 |
|  | Regularly (Weekly) | Ref |  |  |  |
| Recommendations to others | Very unlikely | .967 | .376 | 2.486 | .945 |
|  | Unlikely | .684 | .204 | 2.292 | .538 |
|  | Somewhat likely | 1.477 | .551 | 3.959 | .438 |
|  | Likely | 1.515 | .729 | 3.149 | .266 |
|  | Very likely | Ref |  |  |  |
| Future MH use | Very unlikely | .898 | .572 | 1.411 | .642 |
|  | Unlikely | .998 | .634 | 1.572 | .994 |
|  | Somewhat likely | 1.013 | .639 | 1.604 | .957 |
|  | Likely | .791 | .503 | 1.245 | .312 |
|  | Very likely | Ref |  |  |  |
| MH service type preference\ ^first^ choice | Blended | 2.423 | 1.547 | 3.794 | **<.001** |
|  | Group therapy off-campus | 1.351 | .649 | 2.809 | .421 |
|  | Group therapy on campus | 1.086 | .723 | 1.630 | .691 |
|  | Individual therapy off-campus | 1.573 | 1.163 | 2.126 | **.003** |
|  | Individual therapy on campus | Ref |  |  |  |

*Denotes statistically significant variable
