# Supplementary material for: Awareness, utility and preferences of campus-based mental health services at tertiary institutions in Harare, Zimbabwe: A cross-sectional study
Source: PLOS Glob Public Health. 2026 May 6;6(5):e0005107. doi: 10.1371/journal.pgph.0005107 (PMC13148715; doi:10.1371/journal.pgph.0005107)
Supplement: S4 Table — (DOCX) [file pgph.0005107.s004.docx]

S4 TABLE: Barriers to access to on-campus mental health services, N=1070

Table 1: Barriers to access to on-campus mental health services, N=1070.

| **Domain** | **Item** | **Strongly disagree, n (%)** | **Disagree, n (%)** | **Agree, n (%)** | **Strongly agree,**  **n (%)** |
| --- | --- | --- | --- | --- | --- |
| Stigma-related barriers | Concern about what my family might think, say, do or feel | 146(13.6) | 190(17.8) | 590(55.1) | 144 (13.5) |
|  | Concern that I might be seen as weak for having a mental health problem | 175(16.4) | 324(30.3) | 390(36.4) | 181(16.9) |
|  | Feeling embarrassed or ashamed | 159(14.9) | 313(29.3) | 414(38.7) | 184(17.2) |
|  | Concern about what my friends might think, say or do | 132(12.3) | 294(27.5) | 456(42.6) | 188(17.6) |
|  | Concern about what students might think, say or do | 164(15.3) | 269(25.1) | 454(42.4) | 183(17.1) |
|  | I am worried about my privacy and confidentiality if I use campus-based mental health services | 113(10.6) | 210(19.6) | 435(40.7) | 312(29.2) |
| Attitudinal-related barriers | Thinking the problem would get better by itself | 152(14.2) | 325(30.4) | 436(40.7) | 157(14.7) |
|  | Wanting to solve the problem on my own | 111(10.4) | 313(29.3) | 450(42.1) | 196(18.3) |
|  | Preferring to get help from family or friends | 128(12.0) | 287(26.8) | 531(49.6) | 124(11.6) |
|  | Dislike of talking about my feelings, emotions or thoughts | 110(10.3) | 270(25.2) | 463(43.3) | 227(21.2) |
|  | Thinking that professional care probably would not help | 178(16.6) | 478(44.7) | 308(28.8) | 106(9.9) |
|  | Having had previous bad experiences with campus-based mental health services | 258(24.1) | 469(43.8) | 243(22.7) | 100(9.3) |
| Instrumental-related barriers | Not being able to afford the financial costs involved | 98(9.2) | 249(23.3) | 524(49.0) | 199(18.6) |
|  | Having no one who could help me get professional care | 124(11.6) | 333(31.1) | 476(44.5) | 137(12.8) |
|  | Being unsure where to go to get professional care | 90(8.4) | 291(27.2) | 539(50.4) | 150(14.0) |
|  | Failing to find time to visit campus-based mental health services because of academic commitments | 105(9.8) | 294(27.5) | 502(46.9) | 169(15.8) |
|  | Being too unwell to ask for help | 170(15.9) | 429(40.1) | 377(35.2) | 94(8.8) |
