# Supplementary material for: Awareness, utility and preferences of campus-based mental health services at tertiary institutions in Harare, Zimbabwe: A cross-sectional study
Source: PLOS Glob Public Health. 2026 May 6;6(5):e0005107. doi: 10.1371/journal.pgph.0005107 (PMC13148715; doi:10.1371/journal.pgph.0005107)
Supplement: S5 Table — (DOCX) [file pgph.0005107.s005.docx]

# S5 Table: Actionable recommendations

Table 1 : Actionable recommendations for research, policy and practice mapped onto the socio-ecological systems.

|  | **Actionable focus for institutional leaders** | **Actionable focus for government / policy** | **Actionable focus for researchers** |
| --- | --- | --- | --- |
| **Individual (Microsystem)** | Improve quality and responsiveness of counselling; provide clear information on services including details about the type and location of services | Set minimum standards for student mental-health care within tertiary settings. | Examine determinants of awareness, utilisation and initial help-seeking. |
| **Interpersonal (Mesosystem)** | Strengthen peer-support structures; formalise referral pathways across academic units, counselling units and external partners. | Support integrated referral protocols between tertiary institutions and public mental-health facilities. | Study interaction effects between peer support, family influence and service use. |
| **Institutional (Exosystem)** | Increase visibility of services; maintain ongoing awareness campaigns; expand preferred modalities (in-person, individual, self-help); review MH workshop quality. | Allocate resources for campus mental-health infrastructure and digital self-help platforms. | Investigate unintended consequences of workshops and institutional messaging.  Explore University policies on CBMHS and potential barriers and enablers that can influence the utilization of CBMHS by university students. |
| **Societal (Macrosystem)** | Embed anti-stigma strategies into campus policy; normalise mental-health literacy across the curriculum. | Develop national guidance for campus mental-health services; regulate training of lay providers. | Analyse cultural norms shaping help-seeking and perceived barriers. |
| **Temporal (Chronosystem)** | Track trends in MHS awareness and utilisation over time; adjust interventions as patterns shift. | Maintain longitudinal surveillance of student mental-health indicators nationally. | Conduct longitudinal studies on evolving preferences, barriers and service trajectories. |
